# Printing-Path-Dominated Anisotropy in FDM-PEEK: Modulation by Build Orientation for Tensile and Shear Performance

**DOI:** 10.3390/polym18010041

**Published:** 2025-12-23

**Authors:** Kui Liu, Wei Chen, Feihu Shan, Hairui Wang, Kai Li

**Affiliations:** 1 Aviation Key Laboratory of Science and Technology on Additive Manufacturing, Beijing 100024, China; liukui104@126.com (K.L.); hufei668@126.com (F.S.); 2 Avic Manufacturing Technology Institute, Beijing 100024, China; 3 Key Laboratory of Microgravity, Institute of Mechanics, Chinese Academy of Sciences, Beijing 100190, China; likai@imech.ac.cn; 4 School of Engineering Science, University of Chinese Academy of Sciences, Beijing 100049, China

**Keywords:** fused deposition modeling, printing path, build orientation, anisotropic mechanical behavior, failure mode

## Abstract

Fused deposition modeling of polyether ether ketone offers distinct advantages for fabricating complex and lightweight structures. Although three principal build orientations theoretically exist for practical 3D engineering components, research on their effects remains limited, especially regarding the influence of the interaction between build orientation and printing path on mechanical performance. This study investigated the tensile and shear properties, as well as the failure mechanisms, of FDM-fabricated PEEK under the coupled effects of build orientation and printing path through mechanical testing, fracture morphology analysis, and statistical methods. The results indicate that the printing path exerts a dominant influence on anisotropic behavior, while the interaction between printing path and build orientation jointly governs the shear failure modes. Under identical printing paths, the elongation at break varied by up to twofold across different build orientations, reaching a maximum of 96%, whereas samples printed with W or T paths exhibited elongations at break below 5%. Although shear and tensile moduli remained largely consistent across build orientations, other mechanical properties demonstrated significant differences. Variations in cross-sectional dimensions induced by build orientation markedly affected tensile performance: the coupled effect of build orientation and printing path was found to render the path repetition frequency a critical factor in determining temperature uniformity within the printed region and the quality of interlayer interfaces, thereby constituting the core mechanism underlying anisotropic behavior. Furthermore, larger cross-sections re-duced tensile modulus but enhanced yield strength and elongation at break, highlight-ing the regulatory role of cross-sectional geometry on mechanical response. Based on these findings, a synergistic optimization strategy integrating printing path, build orientation, and tensile–shear performance is proposed to achieve tailored mechanical properties in FDM-fabricated PEEK components. This approach enables controlled enhancement of structural performance to meet diverse application requirements.

## 1. Introduction

Thermoplastic-matrix composites are seeing growing adoption in demanding fields, thanks to their unique benefits such as reprocessability, weldability, short cycle times, recyclability, damage tolerance, and solvent-free processing [1,2,3]. Polyether ether ketone (PEEK) has been increasingly utilized in advanced manufacturing sectors such as aerospace, automotive, medical, and robotics, owing to its exceptional thermal stability, chemical resistance, and mechanical properties [4,5,6]. However, conventional processing techniques face significant challenges when fabricating components with complex geometries, including difficulties in machining, long production cycles, and material waste, which hinder the fulfillment of efficient and customized manufacturing demands [7,8,9,10]. Fused deposition modeling (FDM) is a representative material extrusion additive manufacturing technology, offers notable advantages such as high design flexibility, rapid processing, and low production costs, demonstrating substantial potential in the fabrication of intricate three-dimensional structures [11,12,13,14,15,16,17]. By integrating the superior properties of PEEK with the process benefits of FDM, the limitations of traditional manufacturing methods can be overcome, providing a viable pathway for the rapid prototyping and customized fabrication of complex parts. This integration has thus been identified as a key strategy for expanding the application scope of PEEK.

In recent years, researchers have conducted comprehensive investigations into the effects of FDM process parameters on the performance of PEEK parts, resulting in significant advancements. Regarding the optimization of process parameters, Billè et al. [18] systematically examined the impacts of nozzle temperature, nozzle speed, and layer thickness on the mechanical properties and surface quality of PEEK components. The findings indicated that nozzle temperature was the dominant factor, with optimal performance achieved at a nozzle temperature of 420 °C, a nozzle speed of 40 mm/s, and a layer thickness of 0.1 mm. Deng et al. [19], utilizing a four-factor, three-level orthogonal experimental design, revealed the combined effects of printing speed, layer thickness, printing temperature, and fill density on tensile performance. It was found that optimal tensile properties were achieved at a printing speed of 60 mm/s, a layer thickness of 0.2 mm, a temperature of 370 °C, and a fill density of 40%. Wu et al. [20] focused on the coupling effect between layer thickness and raster angle, indicating that a layer thickness of 0.3 mm combined with a 0° raster angle effectively enhanced the mechanical response. Arif et al. [21] systematically investigated the influence of printing orientation on mechanical properties, revealing significant anisotropy in FDM-fabricated PEEK parts, with a notable decline in mechanical performance observed in samples printed in the V-90° direction. Rinaldi et al. [9] demonstrated that vertically printed samples exhibited pronounced interlayer brittle fractures. Geng et al. [22] examined the effects of extrusion speed and printing speed on the microstructure of PEEK filaments, discovering that reducing extrusion speed promotes molecular chain orientation and energy storage, thereby decreasing the risk of cavity formation. Conversely, abnormal melt pressure was found to potentially induce structural defects. In the control of the temperature field, the effect of nozzle temperature on warpage deformation during PEEK printing was investigated by Hu et al. [23]. It was found that when the temperature distribution became uniform and approached the glass transition temperature of PEEK, the interlayer melt bonding strength was significantly enhanced, effectively suppressing warpage deformation. Wu et al. [24] examined the influence of chamber temperature and nozzle temperature on forming defects. They reported that the chamber temperature regulated the cooling rate, promoting uniform crystallization and thereby effectively reducing warpage and interlayer delamination. Ding et al. [25] observed that within a nozzle temperature range of 360 °C to 420 °C, PEEK structures exhibited favorable plastic deformation capabilities. Regarding post-processing techniques, Yang et al. [26] studied the effects of chamber cooling and thermal annealing on the crystallization behavior and macroscopic mechanical properties of PEEK. The results indicated that both methods increased the material’s crystallinity, leading to improvements in tensile strength and elastic modulus, although a decrease in elongation at break was also noted. Furthermore, many studies have reported the occurrence of secondary recrystallization during annealing; however, the effect of annealing temperature on the final mechanical properties has been found to be relatively limited [27,28,29,30].

The existing studies have primarily focused on the effects of FDM process parameters on the tensile, flexural, and compressive mechanical properties of PEEK parts. However, systematic investigations into their shear properties remain scarce [31]. Furthermore, considering that practical engineering structures often exhibit complex three-dimensional geometries and that printing orientations theoretically encompass at least three distinct modes, comprehensive studies on the coupled effects of build orientation and printing path on structural performance are still lacking [25,32].

However, a systematic understanding of the coupled effects between the multiple build orientations inherently present in 3D practical components and the complex internal printing paths is still lacking. Specifically, current research fails to clearly determine whether the printing path or the build orientation dominates the final mechanical anisotropy under multi-directional conditions, and how they synergistically govern the interlayer bonding quality, defect distribution, and ultimate failure modes. Accordingly, this study aims to address the following objectives through systematic experimentation. First, it seeks to determine which factor—the printing path or the build orientation—plays a dominant role in the mechanical anisotropy of FDM-PEEK under tensile and shear loading. Second, the work investigates how the coupling effect between these two factors specifically governs the material’s failure mechanisms and fracture morphology. Finally, it aims to identify the key microstructural features, such as porosity and interlayer fusion, that underlie these macroscopic performance differences.

This study systematically investigates the influence mechanisms of printing path and build orientation on mechanical anisotropy of FDM-fabricated PEEK. Diverse combinations of process parameters were designed. Tensile and shear performance tests, fracture morphology analysis, and quantitative statistical methods were employed. The evolution patterns of microscopic defects under different build modes were examined in detail. Their regulatory effects on macroscopic mechanical behavior were also explored. The intrinsic relationship between build orientation and printing path optimization strategies was clarified. This work provides theoretical support for multi-scale structural optimization in FDM processes.

## 2. Materials and Methods

### 2.1. Preparation Method and Variable Design

In this study, high-temperature FDM technology was employed using the INTAMSYS FUNMAT 610HT system and PEEK wire (ES10, Weizi New Material Technology Co., Ltd., Shandong, China). The list of process parameters is presented in Table 1. The single parameter set was not chosen arbitrarily but was deliberately fixed to represent a realistic scenario in an industrial context, where a balance must be struck between print speed, detail resolution, and process stability. A printing speed of 120 mm/s was explicitly selected to target high deposition efficiency, a crucial driver for reducing production time and cost in industrial applications. A multi-stage gradient temperature field strategy was implemented in the thermodynamic control system, allowing for precise configuration of key parameters such as nozzle temperature, build platform temperature, and ambient chamber temperature. A post-treatment condition is divided into unannealed treated PEEK with natural cooling in the chamber and annealed treated PEEK according to the temperature program. The heat treatment process is carried out in a blast drying oven (DHG-9145A, Yiheng Scientific Instrument Co., Ltd., Shanghai, China), with a heating rate of approximately 2 °C/min and a temperature hold tolerance of ±1 °C. The cooling process is carried out slowly to the room temperature in the oven. The subsequent crystallinity mechanical correlation is obtained on the same geometric shape.

**Figure 1:**
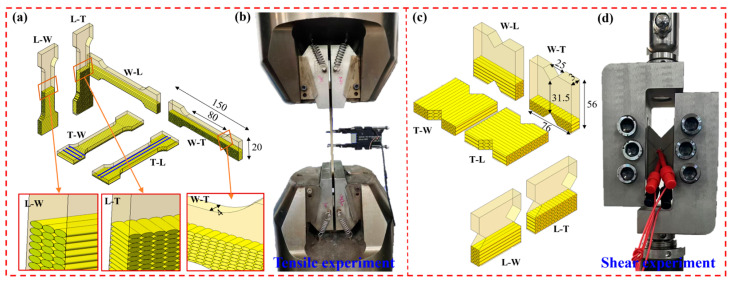
Tensile and shear specimens with different build orientations and printing paths. (**a**) Schematic of build orientation and printing path for tensile specimens. Arrows are localized enlargements. (**b**) Tensile experiment. (**c**) Schematic of build orientation and printing path for shear specimens. (**d**) Shear experiment.

To systematically explore the mechanisms by which build orientation and printing path planning affect the mechanical properties of materials, a controlled variable method was employed in the experimental design. Initially, the build orientation, representing the structural orientation, was defined as follows: horizontal forming was designated as build in the thickness direction (T), lateral forming as build in the width direction (W), and vertical forming as build in the length direction (L). Subsequently, the printing paths were designated as W for the width direction, L for the length direction, and T for the thickness direction. The schematic diagram of the principle of studying different build orientations and printing paths for tensile and shear specimens is shown in Figure 1.

The combinations of these different build orientations and printing paths are shown in Table 2, which includes T-L, T-W, W-L, W-T, L-W, and L-T. This multifactorial design aids in constructing a mapping relationship model between forming process parameters and the mechanical properties of components.

**Figure 2:**
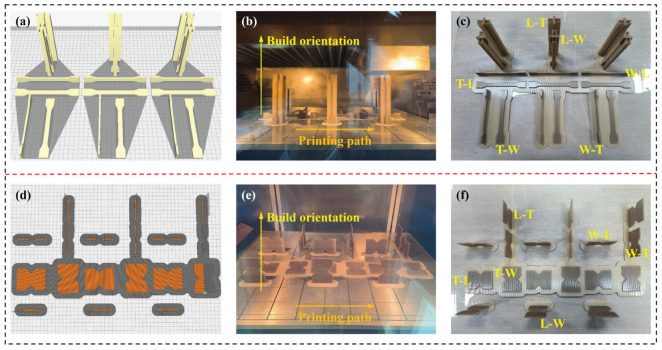
FDM printing process and results of tensile and shear samples. (**a**) Slicing process of tensile samples. (**b**) Printing process of tensile samples. (**c**) Final printed tensile samples. (**d**) Slicing process of shear samples. (**e**) Printing process of shear samples. (**f**) Final printed shear samples.

This research aims to systematically investigate the fundamental effects of build orientation and printing path on the anisotropic mechanical behavior and failure mechanisms of PEEK. Hence, an infill density of 100% topology in the slicing process was used to effectively eliminate the random variable of insufficient infill, thereby ensuring that the observed performance differences can be more directly and reliably attributed to the core process parameters under investigation of build orientation and printing path. The FDM printing process and results of tensile and shear samples are shown in Figure 2, respectively.

**Figure 3:**
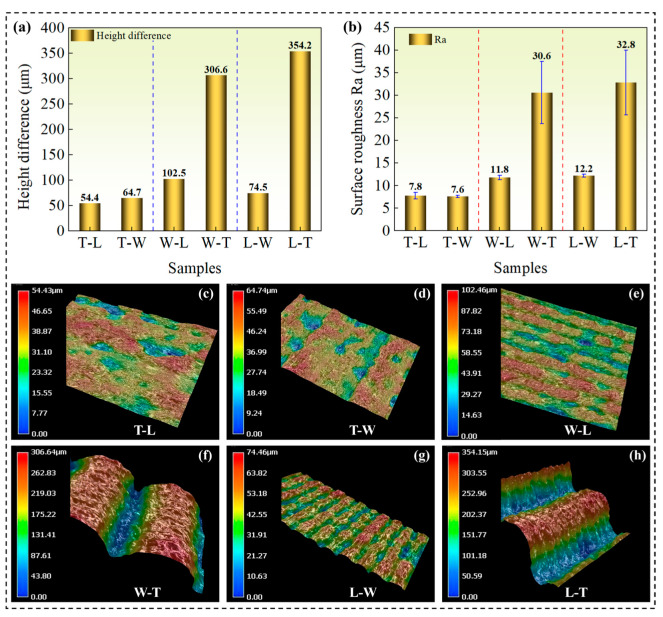
Quantitative analysis of sample surfaces under six different build orientations and printing paths. (**a**) Height difference of samples. (**b**) Surface roughness of samples. (**c**) T-L sample surface. (**d**) T-W sample surface. (**e**) W-L sample surface. (**f**) W-T sample surface. (**g**) L-W sample surface. (**h**) L-T sample surface.

Quantitative analysis of sample surfaces under six different build orientations and printing paths is conducted using VHX-5000 depth-of-field microscope (Keyence Company, Japan) and Taylor Hobson stylus-based surface roughness profiler (Taylor Hobson Company, United Kingdom). As shown in Figure 3a–h, the sample T-L has the lowest height difference value (54.4 μm) and surface roughness Ra value (7.8 μm), while the sample L-T has the highest height difference value (354.2 μm) and surface roughness Ra value (32.8 μm), with a difference of nearly an order of magnitude between the two conditions. This indicates that the build orientation has a decisive impact on surface roughness. The samples T-L, T-W and L-W have a relatively flat surface, with the main morphology being uniformly distributed printed path traces. The samples W-T and L-T exhibit a regular step-like morphology with the rough and uneven surface. The wire accumulation and gaps are obvious. This study quantitatively confirms that in FDM technology, the build orientation is the most important factor affecting surface roughness through the step-like morphology. The printing path determines the microtexture of the surface and the distribution of local defects. By selecting a reasonable build orientation (such as avoiding high-angle surfaces as functional surfaces) and optimizing printing path strategies (such as reducing path twists and increasing the number of contour circles), the surface quality of the sample can be significantly improved.

**Figure 4:**
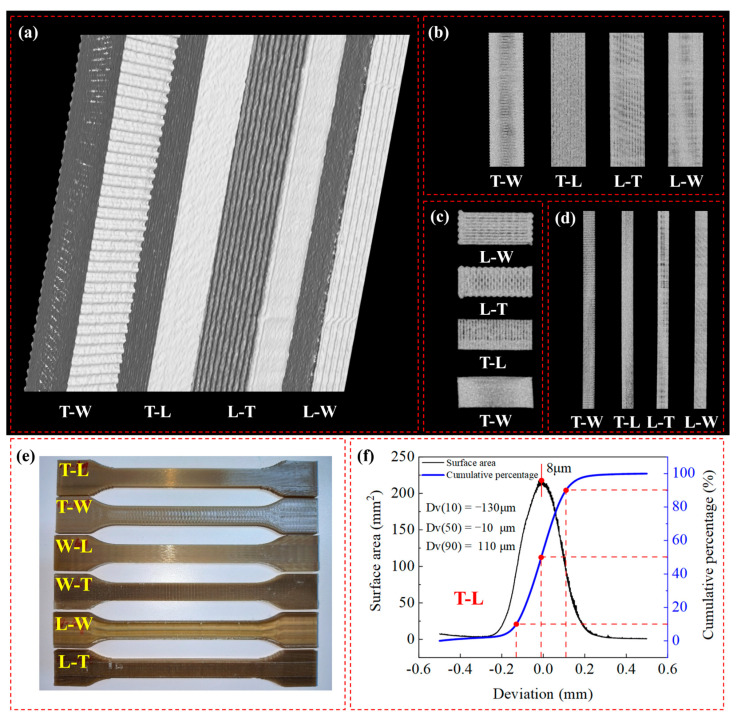
Three-dimensional reconstruction and internal structure from CT scans of representative FDM-fabricated PEEK specimens. (**a**) Isometric view. (**b**) Front view. (**c**) Top view. (**d**) Right view. (**e**) Representative FDM-fabricated PEEK specimens. (**f**) The three-dimensional model reconstruction and quantitative analysis.

To gain an in-depth understanding of the effects of different build orientations and printing paths on the internal structure of FDM-fabricated PEEK specimens, this study employed computed tomography CT to conduct non-destructive testing and three-dimensional reconstruction analysis of the tensile specimens (Figure 4). The micro-focus computed tomography CT system (Model IPT04203C, Granpect Company Limited, Beijing, China) featured a dual-source, single-detector configuration and operated at 450 kV and 160 kV. The CT results revealed significant differences in the internal pore morphology, distribution, and filament fusion state among the specimens, which strongly correlated with their macroscopic mechanical behavior. For specimens printed along the tensile direction (L-path, e.g., T-L shown in Figure 4a–d), the internal pores were fine and isolated, with dense fusion between filaments and no observed continuous gaps between layers. This structural characteristic provides a uniform stress environment for the full extension and sliding of molecular chains, serving as the microstructural foundation for their superplastic deformation. In contrast, for specimens printed perpendicular to or at a large angle to the tensile direction (W or T paths, e.g., T-W, L-W and L-T shown in Figure 4a–d), the CT images clearly showed continuous interlayer gaps extending along the build direction. These inherent weak interfaces became preferred paths for crack initiation and rapid propagation, directly leading to the brittle fracture observed in these specimens. The CT examination visually confirmed from a three-dimensional perspective that the printing path dominates the interlayer bonding quality and defect distribution. Furthermore, the three-dimensional model reconstruction and quantitative analysis based on CT scan results indicated that the dimensional accuracy of the FDM-fabricated PEEK specimens exhibited a clear Gaussian distribution characteristic, with key parameters of DV50 = −10 μm, DV10 = −130 μm, and DV90 = +110 μm. This data reveals that the printed parts overall exhibited a slight negative shrinkage (DV50), indicating good global printing fidelity (Figure 4e,f).

**Figure 5:**
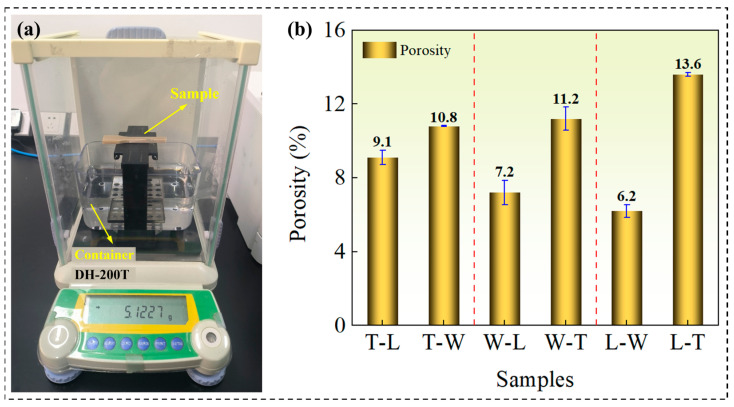
The porosity measurements using the Archimedes water displacement method for the FDM-fabricated PEEK specimens. (**a**) Precision balance. (**b**) Measured values of porosity.

The porosity measurements were conducted using the Archimedes water displacement method. To quantitatively address the porosity, the bulk density (ρ_bulk) of the printed specimens was determined by the water displacement method using a precision balance with a resolution of 0.1 mg. The theoretical density (ρ_theoretical) of fully dense, crystalline PEEK was taken as 1.30 g/cm^3^. The volume porosity (P) was then calculated as: P (%) = [(ρ_theoretical − ρ_bulk)/ρ_theoretical] × 100%. The results of porosity for the different samples are summarized and presented in Figure 5. Although all specimens were printed with a 100% infill density, the apparent porosity measured by the Archimedes water displacement method still ranged from 6.2% to 13.6%, showing significant variation among specimens with different build orientations and printing paths. This result indicates that a 100% infill setting cannot fully eliminate internal porosity within the fabricated parts in the FDM process. The porosity observed in this study reveals the inherent limitations of the FDM process: the actual porosity is not solely determined by the infill density but is predominantly governed by the interlayer fusion quality, which is controlled by the printing path and build orientation.

**Figure 6:**
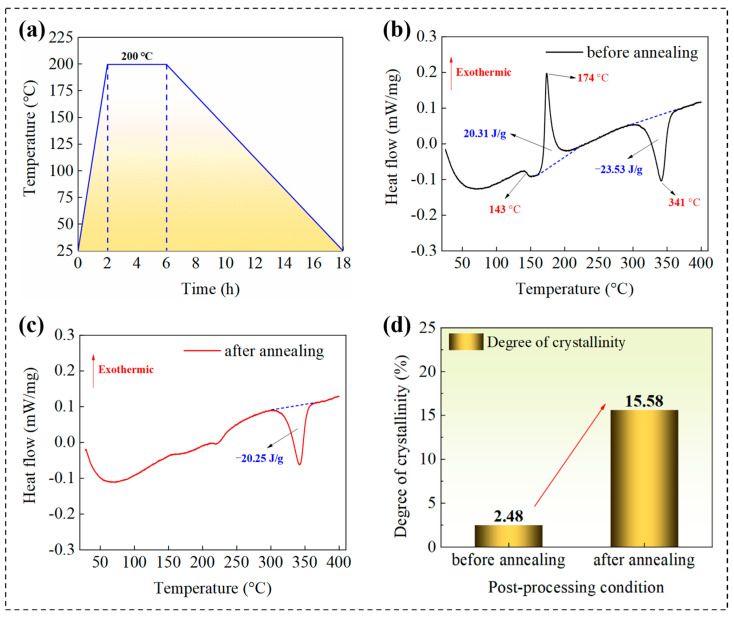
DSC test results before and after annealing treatment of FDM-fabricated PEEK. (**a**) Temperature curve of the annealing process. (**b**) DSC results before annealing treatment. (**c**) DSC results after annealing treatment. (**d**) Comparison of degree of crystallinity before and after annealing treatment.

According to non-isothermal crystallization kinetics, the cooling rate of the process significantly affects the crystallinity of the material, which is manifested as a typical exothermic cold crystallization feature on the DSC curve. Excessive crystallinity results in a reduction in the amorphous region’s volume fraction, leading to a deterioration in the material’s toughness. In this study, the thermodynamic properties of annealed FDM samples were compared with those of unannealed samples under isothermal conditions at 23 ± 0.5 °C using a DSC 404 F3 differential scanning calorimeter (NETZSCH GmbH, Germany). The DSC test results before and after annealing treatment of FDM-fabricated PEEK are shown in Figure 6. It can be observed that the DSC curve of the annealed samples shows a significantly weakened cold crystallization peak, indicating a substantial increase in crystallinity. The samples that were not annealed exhibited a characteristic three-stage phase transition behavior: initially, an endothermic glass transition effect is detected in the temperature range of 140–145 °C (Tg = 143 °C); subsequently, a significant exothermic cold crystallization peak is observed at 171 °C (Tcc), confirming the formation of a metastable amorphous phase during rapid solidification; finally, a sharp and symmetric endothermic melting peak appears at 341 °C (Tm), with a Gaussian distribution revealing the high homogeneity of the crystalline phase structure. As shown in Figure 6d, the enthalpy change of FDM-fabricated PEEK before and after annealing increased from 3.22 J/g to 20.25 J/g. The degree of crystallinity (Xc) using the following established equation was quantified: Xc (%) = [(ΔHm − ΔHcc)/ΔHm^0^] × 100%. ΔHm is the melting enthalpy (J/g). ΔHcc is the cold-crystallization enthalpy (J/g). ΔHm^0^ is the melting enthalpy of a 100% crystalline PEEK sample, for which we have used the widely accepted reference value of 130 J/g. The as-printed specimen without heat treatment exhibited a crystallinity of only 2.45%, characteristic of a metastable amorphous structure formed under rapid cooling. In contrast, after undergoing a standard thermal annealing process, the crystallinity of the specimen increased markedly to 15.58%. The crystallinity results measured by DSC indicate that the heat treatment fundamentally altered the microstructure of the FDM-fabricated PEEK. The unannealed sample retained the metastable amorphous state as a stress-sensitive carrier, which greatly amplified the influence of process parameters on the rearrangement of PEEK molecular chains.

**Figure 7:**
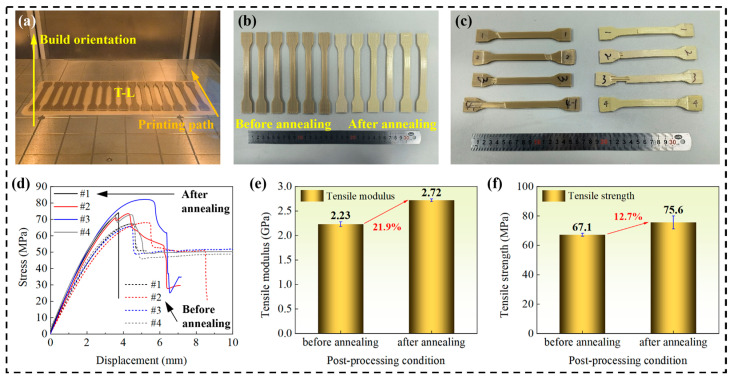
Comparison of mechanical properties of specimens before and after heat treatment. (**a**) Printing process. (**b**) Tensile specimens before testing. (**c**) Tensile specimens after testing. (**d**) Stress–strain curves. (**e**) Comparison of elastic modulus. (**f**) Comparison of tensile strength.

To investigate the influence of heat treatment on the material properties, the tensile performance of FDM-fabricated PEEK specimens was compared before and after oven heat treatment in Figure 7. The results indicate that the heat treatment significantly enhanced the mechanical properties of the specimens. Specifically, the tensile modulus of the heat-treated specimens increased from 2.23 GPa to 2.72 GPa, representing an increase of 21.9%. Concurrently, the tensile strength improved from 67.1 MPa to 75.6 MPa, an increase of 12.7%. This enhancement in performance is primarily attributed to the increased crystallinity and the relief of internal stresses within the material induced by the heat treatment process, which consequently strengthens the intermolecular interactions. The ordered crystalline regions act as physical cross-linking points, more effectively resisting the slippage and deformation of molecular chains, thereby significantly improving the elastic modulus and tensile strength. This aligns perfectly with the observed trends of increased tensile modulus (by 21.9%) and strength (by 12.7%).

### 2.2. Mechanical Testing and Microscopic Characterization

To comprehensively evaluate the properties of the samples, the tensile tests were performed following ISO 527 standards [33], with sample dimensions as shown in Figure 1a, and a controlled tensile speed of 2 mm/min was maintained to acquire data related to tensile properties. For shear testing, the ASTM D7078 standard [34] was employed, using sample dimensions as illustrated in Figure 1c, with a shear speed controlled at 1 mm/min to obtain data pertinent to shear properties. Prior to mechanical testing, the cross-sectional dimensions (width and thickness) of each tensile specimen were precisely measured using a vernier caliper with a resolution of 0.01 mm prior to testing. The width and thickness were measured at least five locations within the gauge length. The average cross-sectional area, calculated from the average measured width and thickness, was used as the initial area for all engineering stress calculations to ensure accuracy and account for process-induced dimensional variations. The reported mechanical trends were based on the actual dimensions of the specimens. The experimental data statistics of six different specimens are listed in Table 3.

To ensure the reliability of the data, a triple-sample validation mechanism was utilized for both experiments. The differences in the effects of the build orientation and the printing path on the tensile and shear properties are studied through the Kruskal–Wallis test. Differences with *p* < 0.05 are considered to be statistically significant. The differences in the effects of the printing path and build orientation on the tensile and shear properties through the Kruskal–Wallis test are shown in Table 4 and Table 5, respectively.

The evaluation of interfacial bonding states and their effects on mechanical properties through high-resolution microscopic observation of fracture surface features in structural materials is a crucial method for elucidating the evolution of mechanical properties. In this study, a SU5000 field emission scanning electron microscope SEM system (Hitachi High-Tech Corporation, Japan) was employed, with the working voltage optimized to 20 kV, to characterize the microstructure of fracture surfaces following tensile and shear failures. During the sample preparation phase, a gold layer was deposited using ion sputtering coating to enhance surface conductivity and optimize imaging resolution. The study focuses on the characterization of the micro-morphology in deformation regions, analysis of crack propagation paths, and a comprehensive exploration of fracture mode mechanisms, systematically revealing the intrinsic relationship between material fracture behavior and microstructural characteristics.

## 3. Results and Discussion

### 3.1. Tensile Performance Analysis

The tensile mechanical responses and microstructural morphologies of six groups of the PEEK specimens were systematically tested. Additionally, statistical analysis of four tensile performance parameters was conducted using the nonparametric Kruskal–Wallis test, as detailed in Table 4 and Table 5.

#### 3.1.1. Analysis of the Influence of Printing Path on Tensile Properties

Under three distinct printing paths, specimens printed along the L path exhibited superior tensile modulus, yield strength, and elongation at break, demonstrating the strongest tensile performance. Statistical analysis (Table 4) revealed significant differences (*p* < 0.05) among the three printing paths in terms of elastic modulus, yield strength, and elongation at break. Specifically, a highly significant difference in elastic modulus was observed between specimens printed along the L and W paths (*p* < 0.01), while specimens printed along the L path showed significant differences (*p* < 0.01) compared to other printing paths in yield strength, and elongation at break. These findings indicate that the printing path exerts a significant influence on the tensile properties of the specimens.

**Figure 8:**
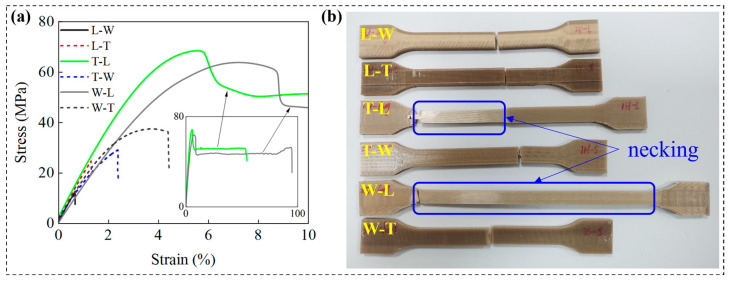
(**a**) Tensile stress–strain curves of different specimens. Arrows are the full tensile stress–strain curve of T-L and W-L specimens. (**b**) Six kinds of specimens after fracture. T-L and W-L specimens exhibit significant necking.

Specifically, specimens printed along the L path (T-L and W-L) exhibited pronounced strain hardening and stress softening behaviors during tensile loading, followed by the formation of characteristic necking after yielding (Figure 8b), indicative of superplastic deformation capability. This behavior is primarily attributed to the highly ordered alignment of PEEK molecular chains along the tensile load direction induced by printing along the L path. The DSC analysis revealed that significant cold crystallization in the as-printed, non-annealed material plays a critical role in enhancing plasticity, especially under conditions of high molecular chain orientation. In this context, the metastable amorphous regions possess elevated thermodynamic driving forces and increased segmental mobility, facilitating the activation of superplastic deformation mechanisms.

**Figure 9:**
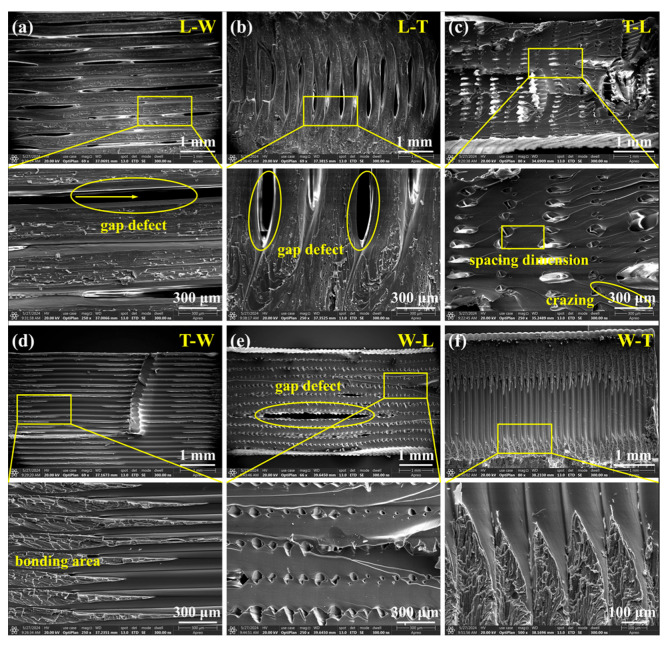
SEM images of the fracture surfaces of the tensile specimens and their magnified images. (**a**) SEM images of L-W specimen. (**b**) SEM images of L-T specimen. (**c**) SEM images of T-L specimen. (**d**) SEM images of T-W specimen. (**e**) SEM images of W-L specimen. (**f**) SEM images of W-T specimen. Circles denote gap defects. Arrow indicates the direction of crack extension.

The SEM images (Figure 9c,e) demonstrated that T-L and W-L specimens possess a continuous and dense microstructure, with uniformly distributed micropores (pore size ≤ 100 μm) accompanied by minimal gap defects, further confirming the structural homogeneity of the material, which contributes to improved tensile strength and ductility. High-resolution images revealed that microcrack initiation around micropores originates from polymer chain slip and orientation during tensile deformation, leading to a transition of stress states from uniaxial to three-dimensional, thereby promoting uniform stress distribution and preventing stress concentration. The propagation and coalescence of these microcracks facilitate the proliferation of shear bands, significantly enhancing elongation at break and overall material toughness.

**Figure 10:**
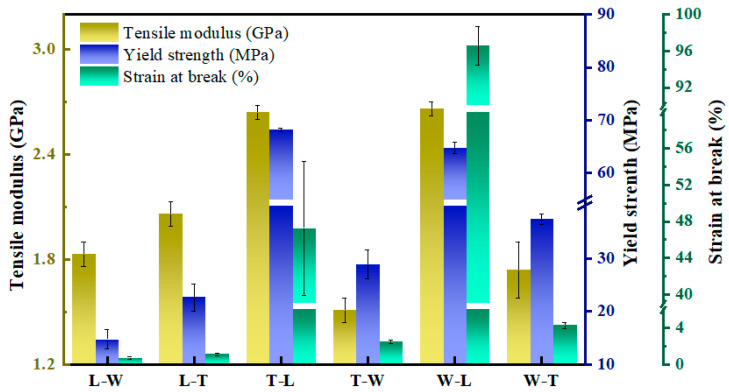
Experimental data statistics of tensile modulus, yield strength and elongation at break of six different specimens.

In contrast, specimens printed along the W path (T-W and L-W) and those printed along the T path (L-T and W-T) exhibited typical brittle fracture modes, with fracture surfaces oriented orthogonally to the load direction (Figure 9b). These samples demonstrated low elastic modulus (≤2.06 GPa), yield strength (≤37.45 MPa), and elongation at break (≤4.28%), as shown in Figure 10. This phenomenon indicates that the ductility-enhancing effect induced by cold crystallization is minimal in these specimens, with insufficient interfacial bonding strength serving as the primary failure mechanism. The underlying cause is attributed to the limited ability of molecular chains to interlayer fusion between stacked layers formed by the FDM technology, which restricts the cooperative movement of PEEK molecular chains. In particular, when the build orientation results in the stacking layer planes being orthogonal to the tensile load direction, interlayer interface failure is prone to occur, leading to a decline in overall plasticity and toughness. The SEM images (Figure 9a,d) reveal that fracture surfaces display pronounced layered morphologies, with continuous gaps present at the interlayer interfaces [35]. High-magnification analysis further illustrates the characteristics of crack propagation: the central regions of the fracture surface, aligned with the filament direction, appear smooth and flat, indicating weaker interlayer bonding in these areas; conversely, the edges exhibit rough, river-like cleavage patterns (labeled as bonding areas), suggesting relatively stronger interlayer adhesion at the edges. These morphological features clearly indicate that the failure mode of these four groups of specimens is brittle fracture, further confirming that weakened interlayer bonding significantly deteriorates tensile performance [25,36].

#### 3.1.2. Analysis of the Influence of Build Orientation on Tensile Properties

Significant differences in tensile properties were observed among specimens oriented in three distinct build orientations. Specimens placed along the L orientation exhibited notably inferior yield strength, and elongation at break, indicating a marked decline in tensile performance. Statistical analysis (Table 5) showed no significant difference in tensile modulus across the three build orientations (*p* > 0.05); however, yield strength (*p* = 0.003), and elongation at break (*p* = 0.002), all demonstrated statistically significant differences. Further multiple comparisons within groups revealed that specimens oriented along the L direction exhibited significantly higher yield strength and elongation at break compared to other orientations (*p* < 0.05). These results confirm that build orientation exerts a significant influence on tensile properties.

**Figure 11:**
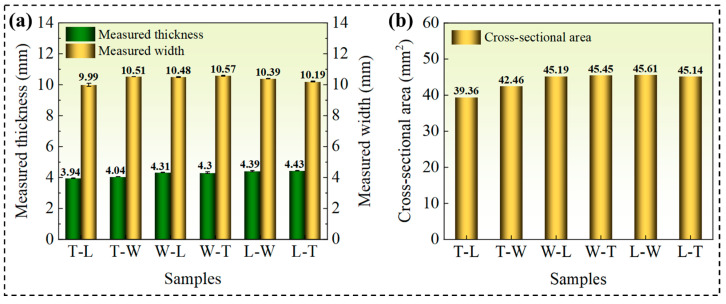
(**a**) Measured dimensions of thickness and width. (**b**) True cross-sectional areas of tensile specimens.

Quantitative analysis revealed that under the L-path printing condition, T-L and W-L specimens exhibited similar filament structures, with elastic moduli of 2.64 GPa and 2.66 GPa, and yield strengths of 68.18 MPa and 64.79 MPa, respectively. However, a significant difference was observed in elongation at break, measuring 46.25% for T-L and 96.60% for W-L—approximately twice that of T-L. The pronounced increase in elongation at break for the W-L specimens is primarily attributed to their relatively small printing cross-sectional area, as listed in Table 6 and Figure 11, which shortens the interlayer reciprocal printing time, resulting in a more uniform overall temperature distribution that promotes effective interlayer fusion [37,38]. Furthermore, high-resolution SEM images (Figure 9c,e) revealed that adjacent filament pores are spaced approximately 300 × 100 μm apart, indicating that the originally circular filaments deform into an elliptical shape during printing due to thermal flow, as illustrated in the enlarged view of Figure 9a. This filament deformation enhances interlayer bonding tightness to a certain extent. These findings demonstrate that build orientation influences tensile performance through variations in cross-sectional geometry.

Similarly, the W-T and L-T specimens exhibited comparable filament microstructures, with elastic moduli of 1.74 GPa and 2.06 GPa, yield strengths of 37.45 MPa and 22.68 MPa, and elongations at break of 4.28% and 1.14%, respectively. The T-W and L-W specimens also displayed similar filament structures, with elastic moduli of approximately 1.51 GPa and 1.83 GPa, yield strengths of about 28.9 MPa and 14.72 MPa, and elongations at break of 2.51% and 0.73%, respectively. Despite these microstructural similarities, significant differences were observed in elastic modulus, yield strength, and elongation at break among these specimens. This phenomenon is primarily attributed to the smaller printing cross-sectional areas in the W-T and L-W printing processes, which reduce the interlayer reciprocal printing time, leading to a more uniform overall temperature distribution and thereby enhancing interlayer bonding tightness. The SEM images (Figure 9b,f) further support this conclusion by showing that the fracture cross-section of L-T specimens is rougher than that of W-T specimens, suggesting stronger interlayer bonding; similarly, the fracture cross-section of L-W specimens is rougher than that of T-W specimens (Figure 9a,d), indicating superior interlayer adhesion. These findings confirm that build orientation influences tensile performance through variations in cross-sectional geometry.

### 3.2. Shear Performance Analysis

The shear mechanical responses of six groups of PEEK specimens were systematically evaluated. Statistical analysis of four shear performance parameters was conducted using the nonparametric Kruskal–Wallis test, as summarized in Table 4 and Table 5.

#### 3.2.1. Analysis of the Influence of Printing Path on Shear Performance

Specimens printed along the T path (L-T and W-T) demonstrated superior performance across multiple shear-related mechanical parameters, including shear modulus, ultimate shear strength, 0.2% offset shear strength, and ultimate shear strain. Statistical analysis (Table 4) indicated significant differences among the three printing paths for these parameters, with *p*-values of 0.002 (<0.01) for shear modulus, 0.029 (<0.05) for offset shear strength, 0.008 (<0.01) for ultimate shear strength, and 0.035 (<0.05) for ultimate shear strain. Further intra-group comparisons revealed that the shear modulus significantly differed between specimens printed along the thickness path and those printed along the other two paths (*p* < 0.05). Additionally, offset shear strength and ultimate shear strength showed significant differences between specimens printed along the length path and those along the thickness path (*p* < 0.05). These results demonstrate that build orientation has a substantial impact on shear performance metrics.

**Figure 12:**
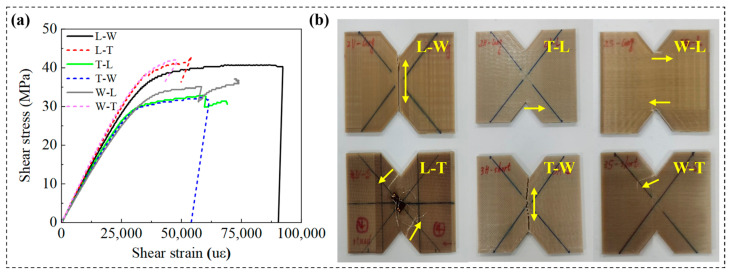
(**a**) Shear stress–strain curves of different specimens. (**b**) Specimens after fracture. Arrows show the fracture patterns and its locations.

Specifically, the L-T and W-T specimens exhibited superior shear performance, with shear crack propagation occurring along oblique paths at angles between 30° and 45° (Figure 12b). This behavior is primarily attributed to printing along the thickness path, which involves shorter printing paths and increased reciprocal printing cycles, resulting in a more uniform temperature distribution within the printing region [39]. Additionally, this orientation increases the number of printed filaments within the shear plane, thereby enhancing interlayer interface bonding strength and effectively improving the material’s shear resistance.

**Figure 13:**
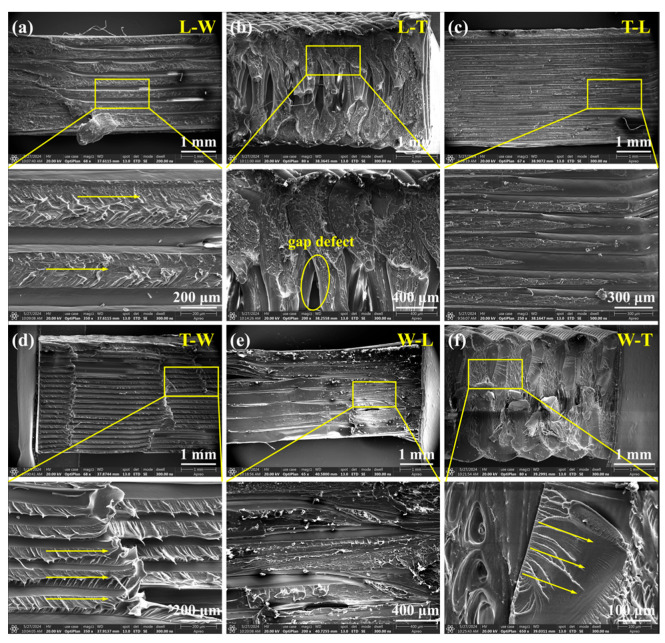
SEM images of the fracture surfaces of the shear specimens and their magnified images. (**a**) SEM images of L-W specimen. (**b**) SEM images of L-T specimen. (**c**) SEM images of T-L specimen. (**d**) SEM images of T-W specimen. (**e**) SEM images of W-L specimen. (**f**) SEM images of W-T specimen. Circles denote gap defects. Arrow indicates the direction of crack extension.

High-resolution SEM observations (Figure 13b,f) revealed the presence of multi-level cracks and tear ridge features along the intralayer interfaces, indicating complex three-dimensional stress redistribution within the material and further corroborating the proposed mechanism. In contrast, specimens printed along the W and L paths exhibited greater variability in shear performance metrics without clear regularity. Failure morphologies shown in Figure 12b indicate that T-W and L-W specimens experienced fractures propagating through the width direction, with crack paths highly aligned with the shear load direction. This behavior is attributed to the alignment of the width printing path with the shear loading axis and the weak interlayer bonding strength at the printing path interfaces, which facilitates preferential crack propagation along interlayer interfaces. The SEM images (Figure 13a,d) reveal pronounced layered fracture morphologies in L-W and T-W specimens, with high-resolution images further disclosing intralayer tearing and interlayer delamination features. This phenomenon primarily results from the filament orientation in T-W and L-W specimens being parallel to the shear plane, reducing interlayer shear resistance and promoting continuous crack propagation along the interlayer interface [31]. Additionally, crack propagation in T-L and W-L specimens exhibited horizontal orientations ranging from 0° to 10°, attributed to the orthogonal relationship between the L printing path, interlayer interfaces, and the shear load direction. In these cases, shear cracks preferentially propagate along the weaker interlayer interfaces. The SEM images (Figure 13c,e) clearly show distinguishable interlayer interfaces with rough fracture surfaces and progressive crack growth along the interfaces, reflecting significant three-dimensional stress redistribution. These observations demonstrate that printing path exerts a significant influence on shear failure modes.

#### 3.2.2. Analysis of the Influence of Build Orientation on Shear Performance

Among the three build orientations, specimens oriented along the L direction exhibited significantly superior performance in ultimate shear strength, 0.2% offset shear strength, and ultimate shear strain. This performance advantage is primarily attributed to the compression of printed filaments along the L direction during the printing process, which enhances interlayer fusion and thereby improves the material’s shear properties. Statistical analysis (Table 5) indicated no significant differences in shear modulus among the three build orientations (*p* > 0.05); however, significant differences were observed in 0.2% offset shear strength (*p* = 0.005 < 0.01), ultimate shear strength (*p* = 0.005 < 0.01), and ultimate shear strain (*p* = 0.035 < 0.05) across different orientations. Further intra-group comparisons revealed significant differences in offset shear strength and ultimate shear strength between specimens oriented along the length and thickness directions (*p* < 0.05). These findings demonstrate a pronounced anisotropy in the shear strength of the FDM-fabricated PEEK materials with respect to build orientation, particularly in ultimate and offset shear strengths.

**Figure 14:**
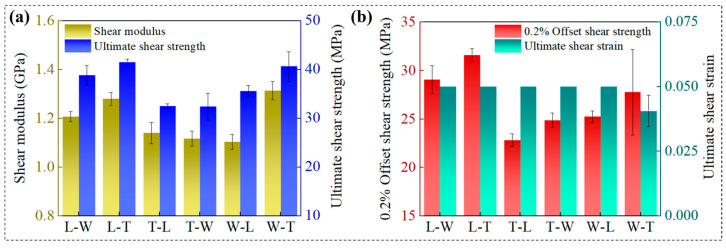
Shear experimental data statistics of 6 different specimens. (**a**) Shear modulus and shear strength of six specimens. (**b**) 0.2% offset shear strength and ultimate shear strain of six specimens.

Quantitative analysis demonstrated that under fixed printing path conditions, the filament structural characteristics of T-L and W-L specimens were similar, exhibiting shear moduli of 1.14 GPa and 1.10 GPa, ultimate shear strengths of 32.50 MPa and 35.53 MPa, and 0.2% offset shear strengths of 22.83 MPa and 25.23 MPa, as shown in Figure 14, respectively. Similarly, W-T and L-T specimens showed comparable filament structures, with shear moduli of 1.31 GPa and 1.28 GPa, ultimate shear strengths of 40.63 MPa and 41.53 MPa, and 0.2% offset shear strengths of 27.77 MPa and 31.60 MPa, respectively. No significant differences in shear performance were observed between these paired groups, indicating that build orientation does not affect shear performance metrics when printing occurs along either the L or T paths. Figure 12 illustrates progressive damage behavior across all four specimen types. The SEM images (Figure 13) revealed highly similar fracture morphologies between T-L and W-L specimens, as well as consistent fracture features between W-T and L-T specimens. These observations confirm that build orientation does not significantly influence shear failure behavior under printing conditions aligned with the L or T paths.

Similarly, under printing conditions oriented along the W path, the filament structural characteristics of the T-W and L-W specimens also exhibited similarities; however, their shear moduli were measured at 1.12 GPa and 1.21 GPa, ultimate shear strengths at 32.4 MPa and 38.8 MPa, and offset shear strengths at 24.87 MPa and 29.07 MPa, respectively, indicating significant differences in shear performance between the two groups. This disparity primarily arises from the differing directions of filament compression: although both printing paths are parallel to the interlayer interface, the filaments in the T-W specimens are compressed along the thickness direction, whereas in the L-W specimens, compression occurs along the length direction. The latter promotes enhanced interlayer fusion, thereby improving shear performance. The SEM fracture analysis revealed that both specimen types exhibited through-thickness damage; however, differences in fracture tearing features along the filament direction were observed. These results indicate that, under W path printing conditions, build orientation significantly affects shear performance metrics but does not substantially influence shear failure behavior.

This study systematically elucidated the pronounced anisotropic mechanical behaviors and underlying failure mechanisms of the FDM-fabricated PEEK arising from the coupled effects of printing path and build orientation through mechanical testing, fracture morphology analysis, and statistical evaluation. Experimental results demonstrated that while shear modulus and tensile modulus exhibited no significant statistical differences across the three build orientations, other tensile and shear performance metrics showed marked variations. Conversely, all tensile and shear parameters varied significantly among the three printing paths, underscoring the dominant influence of printing path on the mechanical properties of the PEEK specimen.

Regarding tensile performance, the coupling effect of build orientation and printing path primarily manifested through variations in interlayer bonding quality and printed cross-sectional dimensions. When printed along the L path, the PEEK molecular chains aligned with the tensile load direction exhibited highly ordered arrangements and enhanced chain segment mobility, facilitating exceptional superplastic deformation capabilities. This resulted in ductile fracture modes with elongations at break reaching up to 96%. In contrast, specimens oriented along the L direction but printed via W or T paths suffered from insufficient interfacial bonding strength, leading to significant deterioration in tensile properties characterized by brittle fracture and elongations below 5%, consistent with earlier studies [37,40,41]. Moreover, differences in printed cross-sectional dimensions induced by build orientation substantially affected tensile behavior: as cross-sectional size increased, tensile modulus tended to decrease, whereas yield strength and elongation at break improved, reflecting the regulatory role of cross-sectional geometry on mechanical response.

In terms of shear performance, build orientation exerted negligible influence on shear metrics when printing occurred along L or T paths, indicating the predominant role of printing path in governing shear behavior. However, under W-path printing conditions, build orientation significantly affected shear properties, revealing its dominant effect in this context. Shear failure modes were closely correlated with printing path: cracks propagated perpendicular to the shear load direction in L-path specimens, exhibited through-thickness failure aligned with shear load in W-path specimens, and developed obliquely along the shear load direction in T-path specimens. These observations highlight the modulation of crack propagation trajectories by interlayer interfaces and printing paths.

Based on these findings, we reveal that, under the coupled effects of placement and printing paths, the frequency of path repetition is inferred to be a critical factor influencing the thermal history, as qualitatively reflected in the color uniformity of the printed specimens. Accordingly, a synergistic optimization strategy integrating printing path, build orientation, and tensile–shear performance is proposed. For structural regions demanding superior tensile properties, printing paths aligned with the tensile load direction should be prioritized, coupled with build orientation adjustments to optimize printed cross-sectional dimensions, thereby enhancing modulus and fracture toughness. Conversely, for areas subjected to high shear demands, printing paths orthogonal to the shear load direction and aligned along the shortest path should be selected, with build orientations configured to render printing paths perpendicular to the shear plane, thus improving shear strength and controlling crack propagation directions.

## 4. Conclusions

This study systematically investigates the anisotropic mechanical behavior and failure mechanisms of FDM-fabricated PEEK components under the coupled influence of printing path and build orientation, through integrated mechanical testing, fracture morphology analysis, and statistical evaluation. The main conclusions are as follows:

The printing path exerts a more substantial influence than build orientation on the mechanical properties of FDM-fabricated PEEK. It significantly affects all evaluated tensile and shear parameters, whereas build orientation shows no statistically significant effect on tensile or shear modulus but does influence other strength and ductility metrics.

Tensile performance is primarily governed by interlayer bonding quality and cross-sectional geometry. Printing along the length (L) path promotes molecular chain alignment along the loading direction, enabling superior superplastic deformation and enhanced fracture toughness. Conversely, shear behavior is predominantly controlled by the printing path itself, with failure modes and strength directly correlated to path orientation relative to the applied load. The influence of build orientation on shear properties is secondary and becomes notable only under specific path conditions, such as the width (W) path.

A novel design strategy is proposed for tailoring mechanical performance by simultaneously optimizing printing path and build orientation according to specific loading conditions. For components bearing tensile loads, aligning the printing path with the loading direction and adjusting build orientation to control cross-sectional dimensions are recommended to improve strength and ductility. For shear-critical applications, selecting paths orthogonal to the shear plane and minimizing interlayer weaknesses through orientation control can enhance performance. This approach facilitates the precision manufacturing of high-performance, application-specific PEEK components using FDM.

## Figures and Tables

**Table 1:** Process parameters of FDM-fabricated PEEK.

Nozzle Diameter (mm)	Nozzle Temperature (°C)	Platform Temperature (°C)	Chamber Temperature (°C)	Layer Height (mm)	Printing Speed (mm/s)	Post-Processing Condition (°C)	
						Unannealed	Annealed
0.4	420	123.5	130	0.15	120	Natural cooling in chamber	200 in oven

**Table 2:** Combined form between build orientations and printing paths.

Printing Path	Build Orientation		
	T	W	L
**T**	--	W-T	L-T
**W**	T-W	--	L-W
**L**	T-L	W-L	--

**Table 3:** Experimental data statistics of six different specimens.

Spec-imen	Tensile Properties				Shear Properties				
	Modulus (GPa)	Yield Strength (MPa)	Strain at Break (%)	Fracture Mode	Shear Modulus (GPa)	Ultimate Shear Strength (MPa)	0.2% Offset Shear Strength (MPa)	Ultimate Shear Strain	Damage Mode
L-W	1.83 ± 0.07	14.72 ± 1.81	0.73 ± 0.15	B	1.21 ± 0.02	38.8 ± 1.99	29.07 ± 1.43	0.05	T
L-T	2.06 ± 0.07	22.68 ± 2.57	1.14 ± 0.14	B	1.28 ± 0.03	41.53 ± 0.65	31.6 ± 0.66	0.05	P
T-L	2.64 ± 0.04	68.18 ± 0.30	47.25 ± 7.35	D	1.14 ± 0.04	32.50 ± 0.46	22.83 ± 0.65	0.05	P
T-W	1.51 ± 0.07	28.90 ± 2.70	2.51 ± 0.18	B	1.12 ± 0.03	32.4 ± 2.78	24.87 ± 0.75	0.05	T
W-L	2.66 ± 0.04	64.79 ± 1.11	96.60 ± 2.11	D	1.10 ± 0.03	35.53 ± 1.17	25.23 ± 0.6	0.05	P
W-T	1.74 ± 0.16	37.45 ± 1.05	4.28 ± 0.34	B	1.31 ± 0.04	40.63 ± 3.06	27.77 ± 4.43	0.0411 ± 0.06	P

Here, B represents the brittle fracture. D represents the ductile fracture. T represents the through damage. P represents the progressive damage.

**Table 4:** The differences in the effects of the printing path on the tensile and shear properties through the Kruskal–Wallis test.

	Median (P25, P75)			H	*p*
	L	W	T		
Tensile modulus	2.65 (2.62, 2.68)	1.67 (1.49, 1.86) a	1.94 (1.71, 2.09)	12.81	0.002 **
Yield strength	66.96 (64.38, 68.2)	21.3 (14.42, 29.58) a	30.56 (22.6, 37.71) a	12.316	0.002 **
Strain at break	74.72 (45.46, 96.72)	1.64 (0.65, 2.52) a	2.6 (1.07, 4.42) a	13.316	0.003 **
Shear modulus	1.12 (1.1, 1.15)	1.17 (1.11, 1.21)	1.3 (1.27, 1.33) ab	12.089	0.002 **
0.2% Offset shear strength	24.05 (22.65, 25.43)	26.4 (24.98, 29.63)	31.15 (27.6, 31.7) a	7.092	0.029 *
Ultimate shear strength	33.7 (32.45, 35.68)	36.5 (31.4, 38.55)	41.85 (39.95, 42.4) a	9.565	0.008 **
Ultimate shear strain	0.05(0.05, 0.05)	0.05(0.05, 0.05)	0.048(0.04, 0.05)	6.733	0.035 *

Here, a indicates that there is a statistical difference from the L printing orientation, and b indicates that there is a statistical difference from the W printing orientation. All comparisons have been corrected by Bonferroni. * *p* < 0.05. ** *p* < 0.01.

**Table 5:** The differences in the effect of the build orientation on the tensile and shear properties through the Kruskal–Wallis test.

	Median (P25, P75)			H	*p*
	L	W	T		
Tensile modulus	1.94 (1.82, 2.09)	2.26 (1.71, 2.67)	2.08 (1.49, 2.65)	0.589	0.745
Yield strength	18.1 (14.42, 23.83)	51.15 (37.18, 64.93) a	49.76 (28.23, 68.20) a	11.368	0.003 **
Strain at break	0.98 (0.65, 1.13)	49.72 (4.25, 96.72) a	21.3 (2.43, 49.13) a	12.316	0.002 **
Shear modulus	1.24 (1.2, 1.29)	1.2 (1.1, 1.33)	1.12 (1.11, 1.16)	5.576	0.062
0.2% Offset shear strength	30.65 (28.93, 31.7)	25.55 (24.15, 29.73)	23.75 (22.65, 25.3) a	10.46	0.005 **
Ultimate shear strength	41 (37.68, 41.68)	36.95 (35.1, 42.4)	32.3 (31.4, 33.53) a	10.725	0.005 **
Ultimate shear strain	0.05(0.05, 0.05)	0.048(0.04, 0.05)	0.05(0.05, 0.05)	6.733	0.035 *

Here, a indicates that there is a statistical difference from the L build orientation. All comparisons have been corrected by Bonferroni. * *p* < 0.05. ** *p* < 0.01.

**Table 6:** Measured dimensions and true cross-sectional areas of tensile specimens.

Sample Name	Measured Width W_m_ (mm)						Measured Thickness T_m_ (mm)						Cross-Sectional Area A_m_ (mm^2^)
	W_m,1_	W_m,2_	W_m,3_	W_m,4_	W_m,5_	W_m_	T_m,1_	T_m,2_	T_m,3_	T_m,4_	T_m,5_	T_m_	A_m_
T-L	9.92	9.91	9.91	10.09	10.10	9.99	3.95	3.94	3.94	3.94	3.95	3.94	39.36
T-W	10.51	10.52	10.52	10.49	10.51	10.51	4.03	4.04	4.04	4.04	4.06	4.04	42.46
W-L	10.47	10.49	10.44	10.48	10.50	10.48	4.31	4.32	4.30	4.31	4.31	4.31	45.19
W-T	10.56	10.59	10.55	10.61	10.55	10.57	4.35	4.26	4.22	4.34	4.33	4.30	45.45
L-W	10.38	10.39	10.40	10.39	10.41	10.39	4.43	4.40	4.45	4.32	4.39	4.39	45.61
L-T	10.17	10.24	10.17	10.16	10.23	10.19	4.42	4.43	4.44	4.43	4.45	4.43	45.14

## Data Availability

The original contributions presented in this study are included in the article. Further inquiries can be directed to the corresponding author.

## Abbreviations

The following abbreviations are used in this manuscript:

FDM	Fused deposition modeling
PEEK	Polyether ether ketone
